# Metabolic assessment of the action of targeted cancer therapeutics using magnetic resonance spectroscopy

**DOI:** 10.1038/sj.bjc.6605457

**Published:** 2009-11-24

**Authors:** M Beloueche-Babari, Y-L Chung, N M S Al-Saffar, M Falck-Miniotis, M O Leach

**Affiliations:** 1 Section of Magnetic Resonance, Cancer Research UK and EPSRC Cancer Imaging Centre, The Institute of Cancer Research and The Royal Marsden NHS Foundation Trust, Surrey SM2 5PT, UK

**Keywords:** non-invasive biomarkers, magnetic resonance spectroscopy, metabolism, targeted therapeutics

## Abstract

Developing rational targeted cancer drugs requires the implementation of pharmacodynamic (PD), preferably non-invasive, biomarkers to aid response assessment and patient follow-up. Magnetic resonance spectroscopy (MRS) allows the non-invasive study of tumour metabolism. We describe the MRS-detectable PD biomarkers resulting from the action of targeted therapeutics, and discuss their biological significance and future translation into clinical use.

The increased understanding of the molecular mechanisms underlying oncogenesis has led to contemporary drug discovery programmes being aimed predominantly at signal transduction pathways and molecules that drive cancer initiation and progression (Gibbs, 2000). The specificity of these agents against a particular deregulated protein/pathway creates new possibilities for tailoring treatment to a particular patient depending on the molecular profile and characteristics of the tumour under investigation (Workman and Kaye, 2002).

Many new agents aimed at blocking particular oncogenic targets are now being used in the clinic, with some having already gained regulatory approval for treatment of certain types of cancers. One example is imatinib mesylate (Gleevec, formerly STI571), which was the first mechanism-based drug targeting an oncogenic molecule to gain FDA approval. Imatinib blocks many protein kinases, including fusion proteins BCR-ABL and KIT, and has been approved for the treatment of chronic myelogenous leukaemia and gastrointestinal stromal tumours.

For the clinical development and evaluation of targeted therapies, and because of the cytostatic mode of action of many of these agents, the methods currently used for assessing tumour response, such as volumetric measurements, may not be adequate. Therefore novel functional pharmacodynamic (PD) biomarkers are required for early stage hypothesis testing to report on target inhibition, assess treatment efficacy and ultimately aid in patient management (Workman *et al*, 2006; Banerji *et al*, 2008). Current techniques for PD biomarker analysis in tumours involve taking surgical biopsy samples for analysis by western blot or ELISA-based assays. This approach has its limitations, as the results obtained can depend on the sampling of heterogeneous disease and sample collection and handling procedure, not to mention the risk and discomfort that may be caused to the patient (Workman *et al*, 2006). Patient acceptance usually limits biopsies to a subset of patients who might also have accessible disease, and generally precludes multiple sampling during treatment. Another approach is to measure samples from the plasma, which provides a source for many markers such as circulating tumour cells, proteins, metabolites and so on. However, this technique suffers from low sensitivity of detection, at least for proteins, and as with tumour biopsies, measurements can be confounded by variability in sample collection, handling and storage, and as a result, the quality of data is often compromised (Hanash *et al*, 2008). The use of normal tissue surrogates for therapeutic action (e.g., hair follicles) has the disadvantage that it may not reflect the molecular expression of the targeted pathway present in tumours.

Non-invasive imaging end points have a distinct advantage in this regard, and are being developed for assessing PD markers of drug activity (Workman *et al*, 2006). There are various non-invasive imaging techniques that can report on tissue function and metabolism, including positron emission tomography (PET), ultrasound, magnetic resonance imaging (MRI) and magnetic resonance spectroscopy (MRS), with each providing a different set of readouts (e.g., metabolism, proliferation, cellularity and so on). This review focuses on MRS as a method for metabolic imaging of PD biomarkers.

## Principle and applications of MRS

Certain atomic nuclei, for example, ^1^H, ^31^P, ^13^C and ^19^F, possess a magnetic property known as the ‘spin’. Metabolites containing these nuclei are detected by means of the interaction of the radio-frequency electromagnetic field with the spin of these nuclei in a strong magnetic field. The separation of resonance frequencies from a chosen reference frequency is termed as the ‘chemical shift’, and is expressed in terms of the dimensionless unit parts per million (p.p.m.) (Gadian, 1995). Metabolites can be identified by their characteristic chemical shift, because of their different chemical structures.

The most common MRS methodologies used to study tumour metabolism are briefly described below (for further details see Chung *et al*, 2006):

- *In vivo* MRS – It can be used to measure the metabolite content of tumours in living animals or patients, repeatedly and non-invasively. Subjects are placed in the bore of the magnet with the tumour positioned in the centre of a surface coil. Localised spectra are acquired and analysed to determine precise chemical shifts and peak integrals. The *in vivo* results are often expressed as ratios of one metabolite to another.
- High-resolution magic-angle spinning spectroscopy (HR-MAS) – It can be used to examine intact cells or small tissue samples (e.g., tumour biopsies). It is invasive but only requires minimal sample preparation. It provides better spectral resolution than *in vivo* MRS and the metabolite levels are often expressed as ratios.
- *In vitro* MRS – It provides a far superior spectral resolution to either HR-MAS or *in vivo* MRS but is invasive and more time consuming in sample preparation than HR-MAS. Cell or tumour samples are first extracted by perchloric acid or methanol/chloroform dual-phase methods, in which water-soluble metabolites and/or lipid metabolites can be obtained. A reference compound with a known concentration is added to the extracts for metabolite quantitation and chemical shift calibration. The metabolite levels are expressed as concentrations.

Magnetic resonance spectroscopy offers a non-invasive means to monitor cell and whole-tissue biochemistry, as it can detect several metabolites in one single measurement and without earlier specification or selection. Magnetic resonance spectroscopy measurements generally include MRI scans that guide localisation of MRS signals to a particular region. They can also provide a wide range of anatomical and functional information as part of the same investigation.

^1^H-MRS has the highest sensitivity and can detect many metabolites including lipids, creatine/phosphocreatine (PCr), glycolytic intermediates such as glucose, glutamine/glutamate and lactate, in addition to choline-containing compounds such as phosphocholine (PC) and glycerophosphocholine (GPC). ^31^P-MRS, on the other hand, has particular value for studies concerned with tissue bioenergetics, pH and membrane turnover, as it can detect the presence of bioenergetic metabolites such as nucleotide triphosphates (NTPs), PCr and inorganic phosphate (Pi), in addition to membrane phospholipid metabolites including phosphomonoesters (PMEs), which comprise PC and phosphoethanolamine (PE), and phosphodiesters (PDEs), which comprise GPC and glycerophosphoethanolamine (GPE). ^13^C-MRS is used to monitor the uptake and metabolism of ^13^C-enriched metabolites and serves as a tool for monitoring the fate of the label, as it is incorporated into other metabolic intermediates such as glutamine and lactate in the case of ^13^C-labelled glucose.

High levels of PMEs, PDEs and total choline (tCho) are observed in tumours by *in vivo* ^31^P- and ^1^H-MRS, which are characteristic metabolic features of cancer (Negendank, 1992; Leach *et al*, 1998). These changes are often reversed on successful treatment with chemo- or radiotherapy and are now being explored as biomarkers for tumour diagnosis, staging and clinical response monitoring (Payne and Leach, 2006; Shah *et al*, 2006). High glucose consumption, as well as lactate production, is another characteristic of cancer cells (also known as the Warburg effect) that forms the basis for metabolic imaging by [^18^F]fluoro-2-deoxy-D-glucose-PET. Several studies have shown the potential of ^1^H- and ^13^C-MRS for monitoring glucose metabolism in pre-clinical models of cancer (Aboagye *et al*, 1998; Rivenzon-Segal *et al*, 2002). However, the clinical measurement of this process in humans by MRS requires further development, which means that, so far, the reported clinical use of MRS has focused primarily on investigating phospholipid metabolism.

Several studies have investigated MRS as an imaging tool for detecting PD biomarkers of the action of novel therapies targeted at aberrant signal transduction pathways. Because only a handful of these agents have so far gained FDA approval, and the fact that most are still in pre-clinical development or early-phase clinical testing, most of the MRS studies performed to date have been concerned with pre-clinical cancer models, including cells and tumour xenografts. However, the technology has been demonstrated in a range of diagnostic and response assessment clinical studies (Howe *et al*, 2003; Meisamy *et al*, 2004; Vilanova *et al*, 2009).

In this paper, we review the current applications of MRS to identify metabolic PD biomarkers within the tumour that follow the action of molecularly targeted cancer therapeutics, with reference to some specific examples. We also discuss the biological significance of these biomarkers in terms of drug-induced anti-neoplastic activity and how they can be translated to patient studies.

## MRS-detectable PD biomarkers of targeted cancer therapies

Table 1 summarises some of the MRS studies monitoring response to different classes of targeted therapies. The changes in bioenergetics, glucose and phospholipid metabolism occurring in cancer cells and tumours after treatment are listed.

Magnetic resonance spectroscopy can be used non-invasively to study the mechanism of drug action and to determine whether target modulation can be monitored. An example of this type of application is a study on a choline kinase (ChoK) inhibitor, MN58b (Al-Saffar *et al*, 2006). ChoK is a cytosolic enzyme that catalyses the phosphorylation of choline to form PC, an intermediate in cell membrane synthesis (Podo, 1999).

Treatment with a ChoK inhibitor should result in a reduction of PC synthesis and this hypothesis was tested in colon (HT29) and breast (MDA-MB-231) cancer cells and tumours treated with MN58b. Decreased PC and tCho levels were found in both cell lines after MN58b treatment and correlated with inhibition of ChoK activity and cell proliferation. *In vivo*, MN58b treatment induced tumour growth inhibition, which was associated with significant decreases in PMEs (^31^P MRS) and tCho (^1^H MRS). *Ex vivo* ^31^P-and ^1^H-MRS analyses of MN58b-treated tumour extracts showed a significant reduction in PC when compared with controls, confirming that the decreases in PMEs and tCho observed *in vivo* were because of a decrease in PC (Al-Saffar *et al*, 2006). In agreement with these findings, inhibition of ChoK using RNA interference in human breast cancer cells was also associated with an MRS-detectable decrease in PC (Glunde *et al*, 2005). These studies highlight the role of PC, tCho and PMEs as potential non-invasive biomarkers for ChoK inhibition.

**Figure 1:**
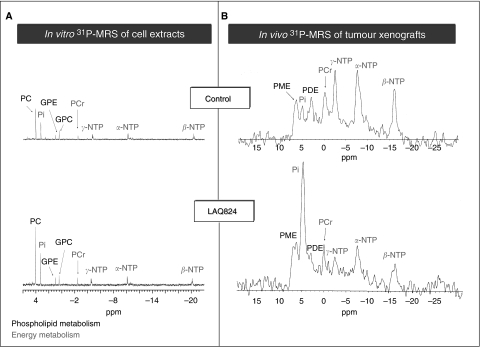
^31^P-MR spectra showing the effect of the HDAC inhibitor LAQ824 on tumour cell metabolism *in vitro* (**A**) and *in vivo* (**B**). Metabolites: PC, phosphocholine; GPC, glycerophosphocholine; GPE, glycerophosphoethanolamine; Pi, inorganic phosphate; PMEs, phosphomonoesters; PDEs, phosphodiesters; PCr, phosphocreatine; NTPs, nucleotide triphosphates.

Another example is the study that was performed using histone deacetylase (HDAC) inhibitors LAQ824 and SAHA. *In vivo* and *in vitro* ^1^H and ^31^P MRS were used to study HT29 cancer cells and tumours following treatment with LAQ824. Significant increases in PC were seen in HT29 cells after LAQ824 (Figure 1A) and SAHA treatment (Chung *et al*, 2008). A rise in PC was also observed in PC3 prostate cancer cells after treatment with an analogue of SAHA (Sankaranarayanapillai *et al*, 2006).

*In vivo*, a significant increase in PME/total phosphorus signal (TotP) was found in LAQ824-treated HT29 xenografts, which correlated with tumour response. A dramatic fall in tumour bioenergetics was also observed, where decreases in intracellular pH, *β*-NTP/TotP and *β*-NTP/Pi, and an increase in Pi/TotP, were found (Figure 1B). Glucose levels were also significantly reduced in drug-treated tumours compared with the vehicle group, whereas lactate levels did not differ significantly.

These changes occurred in parallel with inhibition of tumour growth, histone-3 hyper-acetylation and decreased microvessel density. The increases in PC and PMEs were likely to be associated with HDAC inhibition, whereas the compromised bioenergetics and the fall in glucose content were attributed to the anti-angiogenic effects seen with LAQ824 *ex vivo* (Chung *et al*, 2008). This study showed the value of MRS in identifying and assessing the dual mode of action of LAQ824 in tumours and suggested that the rise in PC and PMEs may be useful as PD markers for tumour response after treatment with LAQ824 or other HDAC inhibitors.

## The meaning and significance of MRS PD biomarkers

As summarised in Table 1, MRS shows many metabolic effects caused by different molecular-targeted agents, some of which could be developed as biomarkers of response in early stage clinical evaluation. These are mainly alterations in phospholipid metabolism (PME, PDE, PC and tCho) and/or glycolysis (lactate) and cellular bioenergetics (NTP, PCr).

Although these metabolic signatures are often different for different classes of agents, they are on the whole consistent with the molecular events triggered after challenge with a particular targeted therapeutic, for example, inhibition of ChoK or activation of phospholipases.

Furthermore, and for a given agent, the effects observed in cell and xenograft models are largely similar, confirming the fact that they are related to the drug's mechanism of action. In the case of the HDAC inhibitor LAQ824, however, decreases in GPC and GPE and cellular energetics were exclusively recorded in tumours, in contrast to the increase in PC, which was observed in cells and tumours alike. This differential effect was attributed to the anti-angiogenic properties of the drug, leading to a reduction in the perfusion and induction of necrotic death in tumours but not in cells.

The metabolic effects induced by the various agents are discussed in more detail in the next two sections.

### Choline phospholipid metabolism

A consistent decrease in PC, PME and tCho levels was observed after inhibition of many molecular targets, including phosphoinositide-3 kinase (PI3K), hypoxia-inducible factor 1*α* (HIF-1*α*), fatty acid synthase (FASN) and BCR-ABL. In contrast, inhibition of heat shock protein 90 (HSP90) or HDAC was associated with an elevation in PC and PME levels.

Phosphocholine is a precursor and a breakdown product of phosphatidylcholine (PtdCho), the major phospholipid component of cell membranes. Phosphocholine can be produced by phosphorylation of choline through ChoK, or from PtdCho hydrolysis directly through PtdCho-specific phospholipase C (PLC) or indirectly through PLD (Podo, 1999). Aberrant choline metabolism has been shown in cancer cells in culture and is also seen in tumour tissue using both *ex vivo* and *in vivo* MRS. Elevated choline levels may be indicative of membrane turnover (Podo, 1999), increased malignant potential (Aboagye and Bhujwalla, 1999) or activation of oncogenic signalling (Ronen *et al*, 2001).

**Figure 2:**
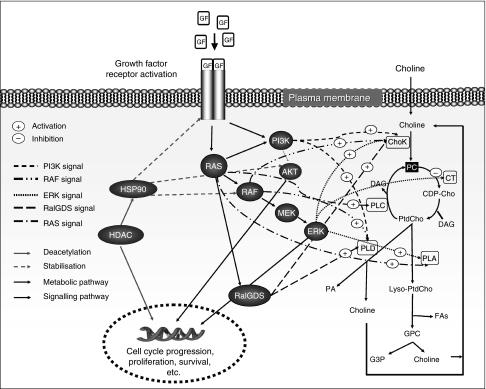
A schematic representation showing links between choline metabolism and some oncogenic signal transduction pathways. Metabolites: CDP-Cho, cytidine diphosphate choline; DAG, diacylglycerol; FAs, fatty acids; G3P, glycerol 3-phosphate; GPC, glycerophosphocholine; Lyso-PtdCho, 1-acyl or 2-acyl-phosphatidylcholine; PA, phosphatidic acid; PtdCho, phosphatidylcholine. Mitogenic signal transduction proteins are shown as black circles and phospholipid metabolic enzymes are shown as white rectangles (modified from Beloueche-Babari *et al*, 2006).

As discussed in Beloueche-Babari *et al* (2006) and in the references therein, signal transduction effectors such as RAS-RAF-MEK-ERK1/2, PI3K/Akt and RalGDS are known to modulate many of the enzymes involved in choline metabolism, including ChoK, phospholipases A, C and D, as well as cytidine triphosphate phosphocholine cytidylyltransferase. Figure 2 shows the choline metabolic pathway and how it is regulated at different points by oncogenic signalling. Hence, modulation of signalling pathways by targeted therapies is expected to affect choline metabolism, leading to changes that may be detectable by MRS.

In line with this, RAS activation in NIH3T3 fibroblasts correlated with an increase in PC levels, which was reversed on treatment with novel RAS signalling inhibitors (Ronen *et al*, 2001). Furthermore, enhanced lipid synthesis after Akt activation was reported and postulated to result from the acceleration of the *de novo* membrane synthesis required to produce increased cell volume (Porstmann *et al*, 2008). This effect was reversed by the inhibition of PI3K/Akt using LY294002 and wortmannin, both of which resulted in a decrease in the PC content detected by MRS (Beloueche-Babari *et al*, 2006). More recently, inhibition of phosphoinositide-specific PLC*γ*1, an enzyme involved in cell invasion and motility, with inducible shRNA, was associated with a fall in PC that was concomitant with reduced cell adhesion and migration. Interestingly, the effect on PC was not observed in cells with a stable knockdown of PLC*γ*1, in which adhesion defects had been by-passed, suggesting that the change in PC is likely to reflect the phenotypic consequences induced by PLC*γ*1 inhibition (Beloueche-Babari *et al*, 2009).

Various studies have focused on ChoK expression/activity as a major regulator of cellular PC, and its link to tumourigenesis (Ramirez de Molina *et al*, 2004; Glunde *et al*, 2005). Hence, the role of ChoK in the MRS-detectable changes in PC after treatment with targeted drugs was investigated. The decrease in PC levels after inhibition of FASN with Orlistat has been attributed to the inhibition of its synthesis as a result of a reduction in ChoK activity (Ross *et al*, 2008). As both FASN (Yang *et al*, 2002) and ChoK (Ramírez de Molina *et al*, 2002) are regulated by MEK-ERK1/2 and PI3K, ChoK could be involved in PC reduction following inhibition of these pathways. Furthermore, regulation of ChoK expression by hypoxia through HIF-1*α* has been reported (Glunde *et al*, 2008). This could be the mechanism underlying the MRS-detected decrease in choline-containing metabolites following inhibition of HIF-1*α* by genetic (Griffiths *et al*, 2002) or pharmacological (PX-478) approaches (Jordan *et al*, 2005).

On the basis of the above, the increase in PC after inhibition of HSP90 or HDAC, agents that were shown to cause anti-tumour effects, is unusual. However, given the number of downstream pathways simultaneously regulated by these targets, the overall metabolic effect is likely to reflect (1) the cellular consequences induced in a particular tumour model (e.g., growth arrest, differentiation, apoptosis); and (2) the balance of interplay between the multiple molecular processes being modulated. Consequently, treatment with these broad-spectrum inhibitors may not necessarily mirror the changes associated with the collective downregulation of downstream signalling pathways using single agents.

As shown in Table 1, a decrease in GPC and GPE was observed by *ex vivo* ^31^P-MRS after inhibition with LAQ824 and PX-478. In contrast, an increase in GPC was detected on exposure to 17-AAG, LY294002 and phenylbutyrate in cell culture, as well as *ex vivo* following treatment with FK866.

Glycerophosphocholine and GPE are released from membrane phospholipids by phospholipase A2 (PLA2) and lysophospholipase. In the case of phenylbutyrate, the rise in GPC levels was associated with mobile lipid accumulation and postulated to occur as a result of PLA2 activation (Milkevitch *et al*, 2005). The indomethacin-induced increase in cellular GPC was also attributed to enhanced membrane turnover through phospholipses (Glunde *et al*, 2006).

Whether 17-AAG and/or LY294002 have similar effects resulting in increased levels of GPC or whether it is a consequence of secondary effects or signalling cascades leading to changes in enzyme activity is still to be investigated.

Conversely, decreases in GPC and GPE were observed *ex vivo* in LAQ824- and PX-478-treated tumours. However, given that both agents show anti-angiogenic effects, these changes may be associated with reduced membrane turnover following necrotic cell death (Jordan *et al*, 2005; Chung *et al*, 2008).

### Glucose metabolism and cellular bioenergetics

^31^P MRS-detectable NTP is mainly derived from ATP, which, together with PCr, is the major donor of free energy in the cell. PCr exerts it effect as a reservoir for the generation of ATP. The phosphate on PCr is donated to ADP to form ATP, to maintain cellular ATP levels, and Pi accumulates in the cell as a result of PCr breakdown. In addition, as cancer cells exhibit increased aerobic glycolysis resulting in an increased rate of glucose uptake and lactate production, ^1^H MRS-detectable lactate may in some cases be used as a tool to evaluate the glycolytic state of cells.

Targeting specific oncogene-driven signalling pathways can result in bioenergetic changes and in alterations in the glycolyic phenotype of cancer cells as shown in Table 1.

Activation of pathways downstream of growth factor receptors e.g. RAS, Akt, HDAC and HSP90, is associated with enhanced glycolysis partly through stabilisation of HIF1-*α* (as reviewed in Gatenby and Gillies, 2007). In response to RAS inhibition, HIF-1*α* levels decrease and glycolysis is inhibited (Blum *et al*, 2005). As expected, inhibition of HIF-1*α* with PX-478 led to a trend of decreased glucose uptake and lactate production in HT-29 tumour xenografts (Jordan *et al*, 2005). Furthermore, inhibition of HSP90 with 17-AAG in HT-29 xenografts resulted in a moderate drop in NTPs (Chung *et al*, 2003), and blockade of HDAC in the same model with LAQ824 resulted in dramatic decreases in PCr, NTP and glucose levels and increased Pi. However, the effects reported, at least in the case of LAQ824, were likely to result from the induced anti-angiogenic effects and the accompanying increase in tumour necrosis (Chung *et al*, 2008).

Both the RAS-RAF-MEK-ERK1/2 and PI3K/Akt pathways are activated downstream of the chronic myeloid leukaemia BCR-ABL fusion protein. Inhibition of the BCR-ABL oncoprotein with imatinib in BCR-ABL-positive leukaemia cells resulted in decreased glucose uptake, lactate production and improved cellular energetics (Gottschalk *et al*, 2004), which were not recorded in their imatinib-resistant counterpart (Kominsky *et al*, 2009). These effects were coupled with translocation of the glucose transporter GLUT-1 from the plasma membrane to the cytoplasm in sensitive but not resistant lines, indicating an inhibition of glucose uptake following treatment with imatinib (Kominsky *et al*, 2009). As aberrant BCR promotes the expression of the pro-glycolytic protein C-MYC (Mahon *et al*, 2003), decreased glycolysis in response to imatinib treatment could be mediated through C-MYC suppression. Inhibition of FASN with Orlistat resulted in a decrease in NTP and PCr levels (Ross *et al*, 2008). FASN can be activated by PI3K and RAF-MEK-ERK1/2 signalling, which suggests that the effects on bioenergetics resulting from Orlistat treatment could be mediated through alterations in either PI3K or MEK-ERK1/2 signalling. However, inhibition with the prototype MEK inhibitor U0126 or the PI3K inhibitor LY294002 did not result in consistent changes in cellular bioenergetics (Beloueche-Babari *et al*, 2005, 2006), which may implicate other pathways in the effects observed with Orlistat.

## Translating MRS studies from the laboratory to the clinic: progress and promise

Changes in glucose and choline metabolism have been described in patients after treatment with various therapies including targeted agents (e.g., imatinib) using PET technology (Heymach *et al*, 2004; Pantaleo *et al*, 2008). PET is a very sensitive method for imaging tumour metabolism. However, and unlike MRS, it requires the administration of radiolabelled substances and cannot discriminate between exogenous and endogenous compounds, or between parent and breakdown products (Payne *et al*, 2006). Hence, the value of MRS as a complimentary tool for metabolic imaging is emphasised.

The clinical use of MRS is well documented, with ^1^H MRS being most widely used because of its high sensitivity, although methods using other nuclei (^31^P, ^19^F and ^13^C) have also been applied (Seddon *et al*, 2003; Arias-Mendoza *et al*, 2006). Increased levels of choline-containing metabolites have been observed *in vivo* by ^1^H-MRS in many cancers including those of the brain, prostate and breast (Leach *et al*, 1998; Howe *et al*, 2003; Vilanova *et al*, 2009). The same technique has also shown that patient treatment with chemotherapy or radiation was associated with a decline in these signals (Leach *et al*, 1998; Meisamy *et al*, 2004).

As indicated above, the MRS studies with targeted cancer therapeutics reported to date have primarily focused on pre-clinical models because very few agents have been approved for use in the clinic. However, this is changing, with more studies being planned as an increasing number of targeted drugs are entering the clinic.

One example of such research is the clinical study that was performed at our Institution using ^31^P-MRS to assess the metabolic PD markers for the action of the HSP90 inhibitor 17-AAG during a phase I trial. The study aimed to correlate molecular markers of HSP90 inhibition (determined from tumour biopsy) with *in vivo* PD changes in tumour phospholipid metabolites. Owing to the nature of the trial (phase I) and the small size of the patient population that was analysed, no firm conclusions could be drawn regarding potential metabolic biomarkers of drug action. However, the study showed the feasibility of the approach in assessing biomarkers of response in future trials (Beloueche-Babari *et al*, 2003). A multicentre comparative study investigating metabolism in non-Hodgkin's lymphomas has demonstrated the potential to implement ^1^H-decoupled ^31^P-MRS at a number of centres (Arias-Mendoza *et al*, 2006). This approach permits the separation of PC and PE peaks within the PME signal, and GPC and GPE peaks within the PDE signal in the *in vivo* ^31^P spectrum, thus allowing changes within individual peaks to be monitored. It remains to be seen whether this methodology could be useful for detecting metabolic biomarkers associated with response to treatment with targeted therapeutics.

These and earlier clinical studies using ^31^P-MRS have highlighted the requirement for lesions to be relatively close to a surface coil for an adequate signal-to-noise ratio to be attained. As this may not always be possible, ^1^H-MRS, which is now well established clinically, also provides an alternative means for evaluating tumour metabolism.

Advances in instrumentation and in the magnetic field strength available on clinical scanners, together with the new fast metabolic imaging techniques using nuclear hyperpolarisation, are helping to increase the sensitivity of MRS and facilitate its clinical implementation. Developing quality assurance protocols and standardising data acquisition methodologies are important aspects that will help evaluate and integrate findings from multiple centres, thereby reducing effort and keeping duplication to a minimum (Leach *et al*, 1994; Evelhoch *et al*, 2005). Continuing progress in this area signifies that the technology could in the near future be available to assess whether the metabolic signatures detectable in pre-clinical models could eventually be translated into robust imaging biomarkers for clinical use.

## Conclusions

Magnetic resonance spectroscopy is showing promise as a non-invasive imaging tool for detecting metabolic biomarkers that are associated with the action of novel targeted therapeutics in pre-clinical models of cancer, and which are consistent with the observed molecular and cellular effects induced by the drugs. Technological advances and strategies for improved data acquisition protocols are helping the clinical implementation of MRS, which will be required for assessing whether the metabolic PD biomarkers observed could be translated into robust radiological tools for use in patient studies with targeted therapies.

## Figures and Tables

**Table 1:** Summary of MRS studies used to assess response to different classes of molecular-targeted therapies in pre-clinical tumour models

**Therapy**	**Molecular target**	**Metabolite changes in cells**	**Metabolite changes *in vivo/ex vivo***	**References**
MN58b	ChoK	↓PC	↓PME, ↓tCho, ↓PC	Al-Saffar *et al* (2006)
17-AAG	HSP90	↑PC, ↑GPC	↑PME, ↑PC, ↑PE, ↓NTP	Chung *et al* (2003)
LAQ824	HDAC	↑ PC	↑PME, ↑PC, ↑PE, ↑choline, ↓GPC, ↓GPE, ↓PCr, ↓NTP, ↑Pi ↓Glucose	Chung *et al* (2008)
SAHA	HDAC	↑tCho, ↑ PC	—	Sankaranarayanapillai *et al* (2006); Chung *et al* (2008)
Phenylbutyrate	HDAC	↑GPC, ↑tCho	—	Milkevitch *et al* (2005)
PX-478	HIF-1*α*		↓tCho, ↓PC, ↓PE, ↓GPC, ↓GPE	Jordan *et al* (2005)
LY294002 & wortmannin	PI3K	↓PC, ↑GPC,↓NTP	^—^	Beloueche-Babari *et al* (2006)
U0126	MEK1	↓PC	—	Beloueche-Babari *et al* (2005)
Orlistat	FASN	↓PC, ↓NTP, ↓PCr		Ross *et al* (2008)
FK866	Nicotinamide phosphoribosyltransferase	—	↓PC, ↑GPC, ↓PCr, ↓NTP, ↓NAD, ↑G6P, ↑F1,6BP, ↑G3P	Muruganandham *et al* (2005)
Indomethacin	COX-1/COX-2	↓PC, ↑GPC	—	Glunde *et al* (2006)
Imatinib	BCR-ABL	↓PC, ↓Lactate, ↓glucose, ↑NTP	—	Gottschalk *et al* (2004); Kominsky *et al* (2009)

Abbreviations: F1,6BP=fructose 1,6-bisphosphate; GPC=glycerophosphocholine; GPE=glycerophosphoethanolamine; G3P=glycerol 3-phosphate; G6P=glucose-6-phosphate; NAD=nicotinamide adenine dinucleotide; NTP=nucleotide triphosphate; PCr=phosphocreatine; PC=phosphocholine; Pi=inorganic phosphate; PME=phosphomonoester.
